# Supplementary material for: Workforce Experiences of a Rapidly Established SARS-CoV-2 Asymptomatic Testing Service in a Higher Education Setting: A Qualitative Study
Source: Int J Environ Res Public Health. 2022 Sep 30;19(19):12464. doi: 10.3390/ijerph191912464 (PMC9566715; doi:10.3390/ijerph191912464)
Supplement: Supplementary file 1 [file ijerph-19-12464-s001.zip › ijerph-1912965-supplementary.pdf]

Supplementary File S1
ATS Staff Interview Topic Guide

YOUR ROLE:

What was your contribution to the service? (e.g., strategy, operations, advisory, academic, administrative, technical, testing delivery, student, or staff support?).

How did you acquire this role?

YOUR TEAM:

Thinking about the team(s) you worked in – had you worked together before?

What is your view towards the way people within the team(s) operated together?

TEAM AND/OR ATS LEADERSHIP:

Thinking about team or service leadership - were you adequately supported in your role?

What is your view of the service leadership? Overall, and within smaller team(s) or area(s).

Were there particular leadership approaches that helped or hindered you in your role?

Did you act in a leadership role yourself, and if so, how was it?

CAREER DEVELOPMENT:

Did your role or contribution to the service generate any immediate personal or professional development opportunities? If so, what were they?

Did you develop any new skills?

How do you feel about your involvement in the service overall? Has it had any implications for you in the medium-to-longer term?
